# Electrophoretic patterns of proteinuria in dogs with Cushing's syndrome

**DOI:** 10.1111/jsap.70034

**Published:** 2025-10-14

**Authors:** J. Milenkovic, T. Francey, A. Schweighauser, J. Howard, S. Kittl, M. Campos

**Affiliations:** ^1^ Department of Clinical Veterinary Medicine Vetsuisse Faculty University of Bern Bern Switzerland; ^2^ Clinical Diagnostic Laboratory Vetsuisse Faculty University of Bern Bern Switzerland; ^3^ Institute of Veterinary Bacteriology Vetsuisse Faculty University of Bern Bern Switzerland

## Abstract

**Objectives:**

To describe the electrophoretic pattern of proteinuria in dogs with naturally occurring Cushing's syndrome. We hypothesised that urine protein electrophoresis in dogs with spontaneous Cushing's syndrome will reveal glomerular proteinuria. We also hypothesised that the severity of proteinuria would decrease during treatment with trilostane.

**Materials and methods:**

This prospective study included dogs with spontaneous Cushing's syndrome (*n* = 19) and healthy dogs (*n* = 10) serving as controls for urine protein electrophoresis. Urinary proteins were evaluated using sodium dodecyl sulphate agarose gel electrophoresis.

**Results:**

Thirteen dogs with Cushing's syndrome had glomerular proteinuria, with high molecular weight bands between 66 and 150 kDa. Three dogs with Cushing's syndrome had mixed glomerular and tubular proteinuria, with low molecular weight bands between 14.3 and 26 kDa, in addition to high molecular weight bands. Three dogs with Cushing's syndrome had a physiological pattern of urine protein electrophoresis and these dogs were non‐proteinuric. Seven dogs were re‐evaluated 4 to 6 months after initiating treatment with trilostane. Before treatment, six dogs were proteinuric and one dog was non‐proteinuric. After the treatment, three dogs were non‐proteinuric and four dogs were proteinuric. Of these, proteinuria decreased in one dog (50%) and increased (up to 70%) in three dogs.

**Clinical significance:**

Dogs with Cushing's syndrome predominantly exhibit glomerular proteinuria. No systematic improvement was observed during trilostane treatment.

## INTRODUCTION

Cushing's syndrome is a frequent endocrine disorder affecting middle‐aged and older dogs and is characterised by clinical and clinicopathological signs of excessive circulating concentrations of glucocorticoids (Caragelasco et al., 2017). Pathological proteinuria, defined as a urinary protein‐to‐creatinine ratio (UPC) greater than 0.5, has been reported in 44 to 75% of dogs with naturally occurring Cushing's syndrome and has been found to persist despite successful treatment of the disease (Lees et al., 2005; Ortega et al., 1996; Smets, Lefebvre, Aresu, et al., 2012; Smets, Lefebvre, Meij, et al., 2012; Waters et al., 1997). There are only a few studies investigating the relationship between hyperadrenocorticism and renal injury, resulting in limited information on the renal origin of proteinuria in these dogs (Caragelasco et al., 2017; Smets et al., 2010; Smets, Lefebvre, Aresu, et al., 2012; Smets, Lefebvre, Kooistra, et al., 2012). One study, investigating urinary markers of renal function, found evidence of alterations in both glomerular and tubular functions in dogs with Cushing's syndrome (Smets, Lefebvre, Kooistra, et al., 2012). Studies evaluating renal histopathology in dogs receiving exogenous glucocorticoids found glomerular changes (Waters et al., 1997) or both glomerular and tubular alterations (Smets, Lefebvre, Aresu, et al., 2012), but similar changes were found in the control dogs in the latter study. However, a study focusing on urine protein electrophoresis in dogs with ACTH‐dependent hyperadrenocorticism (ADHAC) found that proteinuria was mainly associated with low molecular weight (LMW) proteins, indicating tubular rather than glomerular injury (Caragelasco et al., 2017). Furthermore, the tubular proteinuria persisted during 1 to 6 months of medical treatment. However, another study found both low and high molecular weight (HMW) proteins on urine protein electrophoresis in dogs with hyperadrenocorticism, indicating both glomerular and tubular injury (Cavalcante et al., 2013).

Electrophoresis is a useful technique to localise the origin of pathological renal proteinuria. By separating urine proteins based on their size, sodium dodecyl sulphate agarose gel electrophoresis (SDS‐AGE) can be used to qualify urinary proteins as glomerular, tubular or both (Zini et al., 2004). A glomerular pattern of proteinuria observed in SDS‐AGE is a strong indicator of glomerular disease, correlating well with histopathological findings (Hokamp et al., 2018; Zini et al., 2004). A recent study using SDS‐AGE concluded that in proteinuric dogs with ADHAC, proteinuria is mainly of glomerular origin, with about 25% of dogs having a mixed glomerular and tubular proteinuria (Menard et al., 2024). SDS‐AGE of urine proteins warrants further investigation in dogs with Cushing's syndrome.

The objectives of this study were to describe the electrophoretic pattern of proteinuria in dogs with naturally occurring Cushing's syndrome. We hypothesised that dogs with spontaneous Cushing's syndrome have a predominantly glomerular pattern of proteinuria. We also hypothesised that the severity of proteinuria improves during treatment with trilostane.

## MATERIALS AND METHODS

This prospective study evaluated client‐owned dogs with naturally occurring Cushing's syndrome using comprehensive diagnostic protocols, including endocrine testing, blood analysis, blood pressure assessment and urinalysis. Urine protein electrophoresis was performed to assess proteinuria patterns, and treatment response to trilostane was evaluated by changes in clinical signs and measurement of pre‐pill cortisol concentration. Healthy dogs served as controls for urine protein electrophoresis. Informed consent was obtained from all owners.

### Dogs with spontaneous Cushing's syndrome

Client‐owned dogs diagnosed with naturally occurring Cushing's syndrome were recruited between December 2020 and January 2023. The diagnosis was established according to the Agreeing Language in Veterinary Endocrinology (ALIVE) criteria (ESVE, 2021), based on supportive history, the presence of typical clinical and clinicopathological findings, and either a low‐dose dexamethasone suppression test (LDDST) or an ACTH stimulation test indicating hypercortisolism. Cushing's syndrome was further classified as ADHAC or ACTH‐independent hyperadrenocorticism (AIHAC) based on results of the LDDST, abdominal ultrasonography or computed tomography. Additionally, systemic blood pressure was measured using an oscillometric device (SunTech Vet25, Morrisville NC, USA) in accordance with ACVIM guidelines for hypertension (Acierno et al., 2018). Dogs were categorised based on their systolic blood pressure as follows: normotensive (<140 mmHg), prehypertensive (140 to 159 mmHg), hypertensive (160 to 179 mmHg) and severely hypertensive (>180 mmHg) (Acierno et al., 2018). Dogs with other causes of proteinuria, such as infectious disease, dogs with evidence of urinary tract inflammation (urine sediment showing >5 WBCs/high power field) and dogs with positive urinary culture were excluded. Infectious diseases were ruled out in all dogs based on serological tests for *Ehrlichia canis, Borrelia burgdorferi*, *Anaplasma phagocytophilum* and antigen tests for *Dirofilaria immitis* and *Angiostrongylus vasorum*. *Leishmania* sp. serology was performed in dogs that had travelled to endemic areas. Comprehensive diagnostic evaluation was performed in all dogs, including a complete blood count, biochemical analyses, urinalysis, urinary protein‐to‐creatinine ratio (UPC), urine culture and urine protein electrophoresis. All dogs were treated medically with trilostane (Vetoryl, Dechra Veterinary Products Suisse GmbH, Basel, CH). No dog received additional treatment for proteinuria. Urine and blood samples were collected at initial presentation, and urine was resampled 4 to 6 months after initiation of treatment with trilostane. Response to treatment was evaluated based on clinical signs and two pre‐pill serum cortisol concentrations (1 h apart) (Boretti et al., 2018). Pre‐pill cortisol concentrations of 1.5 to 5.0 μcg/dl were considered within target range. Dogs were excluded if they received antibiotics in the 10 days prior to presentation or if they received either angiotensin‐converting enzyme inhibitors or angiotensin‐2 receptor blockers in the 14 days prior to presentation.

### Healthy dogs

Ten apparently healthy dogs from co‐workers were included to assess physiologic urine protein electrophoresis and UPC. Inclusion criteria were the absence of clinical signs of disease, normal physical examination, unremarkable complete blood count, biochemistry and urinalysis, and a negative urine culture.

### Laboratory analyses

All urine samples were obtained by cystocentesis. Measurement of urine total protein and creatinine concentration was performed on the supernatant of urine centrifuged for urinalysis using a commercial benzethonium chloride method for total protein (TPUC3, Total Protein Urine/CSF, Roche Diagnostics) and an enzymatic creatinine assay (CREP2, Roche Diagnostics) on a commercial biochemistry analyser (Cobas C501, Roche Diagnostics). The remaining supernatant was refrigerated at 4°C pending electrophoresis, which was performed within 1 week of sampling. Following the staging system of the International Renal Interest Society (IRIS), proteinuria was defined as UPC > 0.5. Dogs with UPC < 0.2 were classified as non‐proteinuric and dogs with UPC 0.2 to 0.5 were classified as borderline proteinuric (IRIS, Staging of CKD, 2011). Urine protein electrophoresis was performed on SDS‐AGE gels specifically designed for urine protein electrophoresis (Hydragel 5 Proteinurie, Ref 4115, Sebia Swiss GmbH) run on a semi‐automated analyser (Hydrasys 2 Scan Focusing, Sebia Swiss GmbH) following the manufacturer's guidelines. Based on the manufacturer's documentation, the minimum detection level using this method is about 0.15 g/L protein per fraction. Each gel was analysed to determine protein size together with control material (Molecular Mass Control, Sebia Ref 4781, Sebia Swiss GmbH) containing lysozyme (14.3 kDa), triose phosphate isomerase (26.6 kDa), bovine albumin (66 kDa) and human IgG (150 kDa). The stained and dried gels were analysed by visual inspection comparing bands of the samples with bands in the control lane, and by examining the electrophoretogram made by scanning the gels using commercial software (Phoresis Core, Sebia Swiss GmbH). This software creates a curve and calculates the percentage of albumin, LMW proteins and HMW proteins, and multiplies these percentages by the total protein concentration to give an absolute concentration for each of the three fractions. Urinary protein bands were considered HMW if they fell above 66 kDa (albumin band) and LMW if they were below 66 kDa. Urine protein electrophoresis patterns were classified as glomerular when only HMW proteins were present in addition to albumin, tubular when only LMW proteins were present in addition to albumin, and mixed when both HMW and LMW proteins in addition to albumin were observed. A physiological urine protein electrophoresis pattern was defined as either a single band at 66 kDa, corresponding to the albumin band or no detectable bands at all, typically due to total protein concentration below the assay's sensitivity. Dogs with a single band at 66 kDa (corresponding to albumin) in combination with proteinuria were classified as having a glomerular pattern.

## RESULTS

### Dogs with spontaneous Cushing's syndrome

Nineteen dogs diagnosed with spontaneous Cushing's syndrome were included. There were three mixed breed dogs, two Yorkshire terriers, two shih‐tzus, two Chihuahuas and one each of the following breeds: English Bulldog, French bulldog, Border terrier, Newfoundland, Miniature Pincher, Havanese, Welsh terrier, Jack Russel terrier, American Stafford terrier, and dachshund. The median age of dogs was 12 years (min‐max, 6 to 15 years). Eleven dogs were spayed females, six were neutered males and two were intact males. One dog was under treatment for hypertension (amlodipine 0.5 mg/kg q24h) before enrolling in the study. Eight dogs were normotensive, seven dogs were prehypertensive, one dog was hypertensive, and three dogs did not have blood pressure measurements. For the 16 dogs with blood pressure measurements, the median systolic blood pressure was 141 mmHg (min‐max, 117 to 166 mmHg). Sixteen dogs were diagnosed with ADHAC and three dogs with AIHAC. Fifteen dogs (79%) were proteinuric, one dog was borderline proteinuric and three (16%) dogs were non‐proteinuric (Table 1). No dog was azotemic median creatinine concentration, 49.5 μmol/L (min‐max, 25 to 118 μmol/L). Normotensive dogs had slightly higher median UPC (median 1.7; min‐max, 0.11 to 8.36) than prehypertensive/hypertensive dogs (median 0.87; min‐max, 0.11 to 4.41).

**Table 1:** Urinary protein‐to‐creatinine ratio (UPC), and percentages of glomerular, mixed, tubular and physiological pattern of urine protein electrophoresis in 19 dogs with spontaneous Cushing's syndrome (CS) at diagnosis, seven dogs with Cushing's syndrome 4 to 6 months after treatment (CSt) and 10 healthy dogs (H)

Group	N	UPC	Glomerular	Tubular	Mixed	Physiological	UPC < 0.2	UPC 0.2 to 0.5	UPC > 0.5
		Median	*N* (%)	*N* (%)	*N* (%)	*N* (%)	*N* (%)	*N* (%)	*N* (%)
		Min‐max (IQR)							
CS	19	1.25	13 (68)	0 (0)	3 (16)	3 (16)	3 (16)	1 (5)	15 (79)
		0.11 to 8.36 (0.56 to 2.91)							
CSt	7	0.53	4 (57)	0 (0)	1 (14)	2 (29)	2 (29)	1 (14)	4 (57)
		0.09 to 14.11 (0.18 to 6.33)							
H	10	0.08	0 (0)	0 (0)	0 (0)	10 (100)	10 (100)	0 (0)	0 (0)
		0.06 to 0.15 (0.07 to 0.12)							

### Urine electrophoresis in dogs with spontaneous Cushing's syndrome at presentation

**FIG 1:**
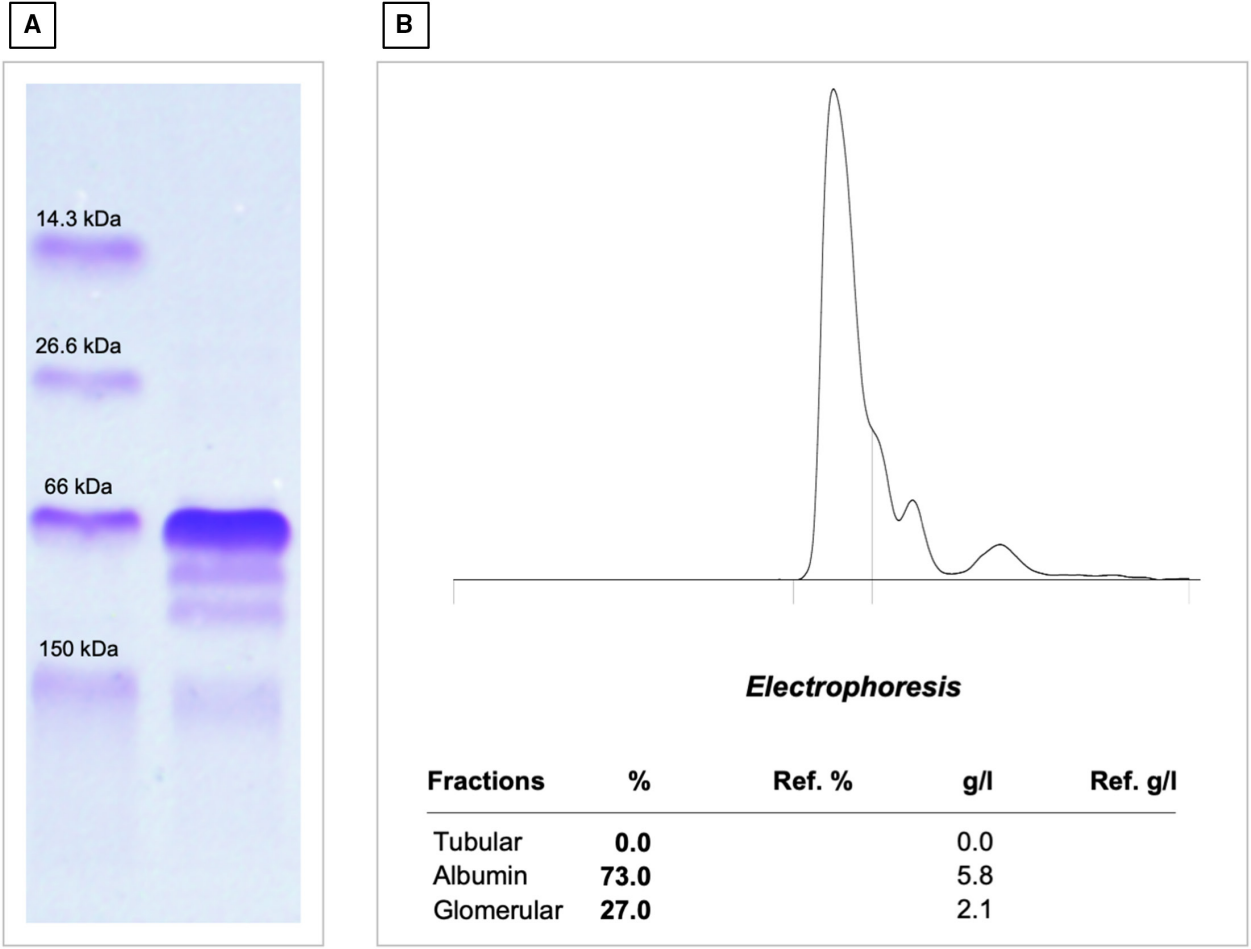
(A) Urine electrophoresis gel of the molecular marker material (left lane) and urine from a dog with Cushing's syndrome before treatment (right lane), showing one band at 66 kDa corresponding to albumin and three bands in the high molecular weight range (two bands between 66 kDa and 150 kDa and one band at 150 kDa). (B) Electrophoretogram of the urine electrophoresis gel of the same dog with Cushing's syndrome before treatment, showing albumin accounting for 73% of the total urine protein, glomerular protein contributing 27% and tubular protein contributing 0.0%. The absolute concentrations were 5.8 g/L albumin and 2.1 g/L glomerular proteins. The UPC of this dog was 8.36 before treatment.

Dogs that were non‐proteinuric had a physiological pattern on urine protein electrophoresis with a single faint band at 66 kDa, except for one dog that had no visible bands. The total urinary protein concentration of this dog was 0.07 g/L, which is below the sensitivity for the detection of protein fractions with the SDS‐AGE method used. The dog that was borderline proteinuric had a glomerular pattern. Of the proteinuric dogs, 12 had a glomerular pattern, while three had a mixed/glomerular pattern. Of the 12 glomerular cases, 11 showed HMW bands and one showed a single albumin band (Table 1). Dogs with a glomerular pattern had 1 to 3 HMW bands and dogs with a mixed pattern also had 1 to 2 LMW bands. HMW bands were observed between 66 and 150 kDa (Fig. 1) and LMW bands were observed between 14.3 and 26.6 kDa. Dogs that had LMW bands included two intact males and one spayed female. One intact male dog had a single LMW band at 26.6 kDa. Although this dog was classified as having a mixed electrophoretic pattern, it should be noted that the band at 26.6 kDa could correspond to arginine esterase, a physiological prostatic protein in intact male dogs. However, this band was no longer present after treatment with trilostane. Another intact male had two LMW bands – one between 14.3 and 26.6 kDa and one at 26.6 kDa. All dogs with AIHAC had glomerular proteinuria.

### Dogs with spontaneous Cushing's syndrome after treatment

**FIG 2:**
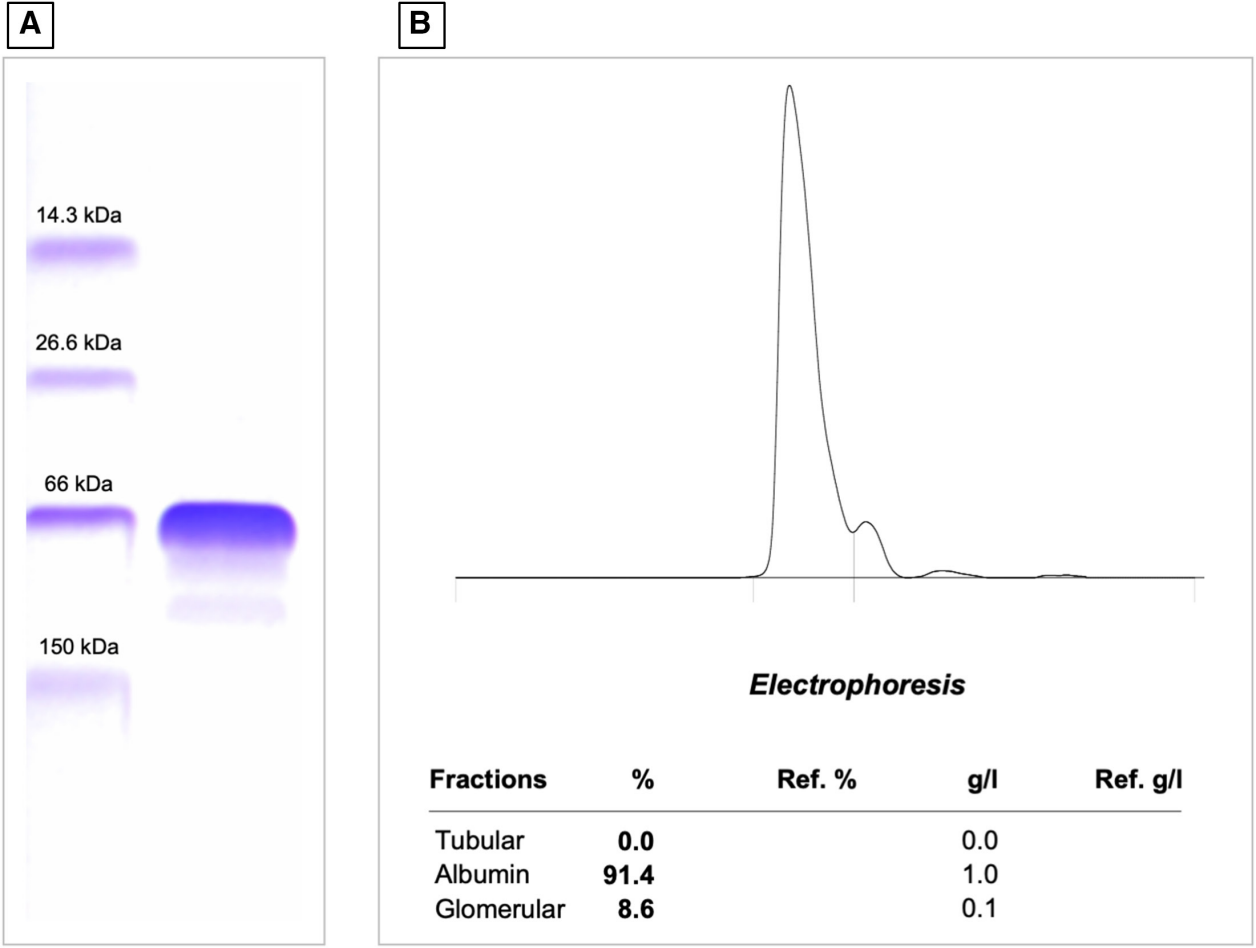
(A) Urine electrophoresis gel of the molecular marker material (left lane) and urine from a dog with Cushing's syndrome 4 to 6 months after trilostane treatment, showing one band at 66 kDa corresponding to albumin and two bands in the high molecular weight range between 66 kDa and 150 kDa. (B) Electrophoretogram of the urine electrophoresis gel of the same dog with Cushing's syndrome 4 to 6 months after trilostane treatment, showing albumin accounting for 91.4% of the total urine proteins, glomerular proteins contributing 8.6% and tubular proteins contributing 0.0%. The absolute concentration of proteins was 1 g/L albumin and 0.1 g/L glomerular proteins. The UPC of this dog was 0.94 after treatment.

During the study, six dogs died: one from cardiac failure, another euthanased after showing neurological signs possibly due to a pituitary tumour, and four dogs from unknown causes. Four dogs were lost to follow‐up, and one dog was excluded at follow‐up due to a urinary tract infection. For the remaining seven dogs, urine protein electrophoresis was repeated 4 to 6 months (median, 4 months; min‐max, 4 to 5 months) after initiating medical treatment with trilostane (trilostane doses at the beginning of treatment: median 1.75 mg/kg/day; min‐max, 1 to 2.6 mg/kg/day, trilostane doses 4 to 6 months after starting treatment: median 1.35 mg/kg/day; min‐max 1 to 2.2 mg/kg/day). Based on clinical signs and physical examination, all seven dogs showed improvement in their Cushing's syndrome. Pre‐pill cortisol concentrations 4 to 6 months after starting medical treatment were: pre‐pill cortisol 1: median, 3.76 μcg/dl (min‐max, 1.86 to 9.45 μcg/dl) and pre‐pill cortisol 2: median, 2.88 μcg/dl (min‐max 1.6 to 11.2 μcg/dl) (*n* = 7) (Table 2). All dogs were included in the study regardless of the results of the pre‐pill cortisol concentration. Two dogs began to exhibit neurological signs, likely attributable to their pituitary mass (ADHAC). After treatment, two dogs were non‐proteinuric, one dog was borderline proteinuric and four dogs were proteinuric. Of the two dogs that were non‐proteinuric after treatment, one dog was non‐proteinuric and one dog was proteinuric prior to treatment. The borderline proteinuric dog after treatment was proteinuric before treatment. Of the 4 dogs with post‐treatment proteinuria, all were proteinuric prior to treatment. Of these, the UPC decreased by 50% after treatment in one dog but increased by up to 70% after treatment in the other three dogs. Four dogs exhibited a glomerular pattern, one dog showed a mixed pattern and two dogs showed a physiological pattern (Table 1, Fig. 2). Of the four dogs with a glomerular pattern after treatment, three had a glomerular pattern prior to treatment and one had mixed proteinuria prior to treatment. The dog that had mixed proteinuria after treatment initially had glomerular proteinuria before treatment. Of the two dogs with no visible bands on urine electrophoresis (except a single band at 66 kDa corresponding to albumin) after treatment, one had no visible bands before treatment, while the other had mixed proteinuria before treatment (Table 2).

**Table 2:** Additional information about seven dogs with Cushing's syndrome 4 to 6 months after treatment with trilostane

Dog number	Breed	Age (years)	Sex	ADHAC or AIHAC	Trilostane dose	Follow‐up time (months)	Pre‐pill cortisol (μcg/dl)	UPC before treatment	UPC after treatment	Pattern of proteinuria before treatment	Pattern of proteinuria after treatment	Clinical status after treatment
2	English bulldog	11	Fc	AIHAC	0.5 mg/kg q 12 h	4	2.31, 2.88	1.25	0.49	Glomerular	Glomerular	Improvement of skin changes, PU/PD resolved, mildly distended abdomen
4	Yorkshire terrier	9	Fc	ADHAC	1 mg/kg q24h	5	3.76, 11.20	8.36	14.11	Glomerular	Mixed	PU/PD and polyphagia resolved, improved alopecia
5	Shih‐tzu	12	Mc	ADHAC	1 mg/kg q 12 h	4.5	5.34 2.69	0.11	0.18	Physiological	Physiological	PU/PD and polyphagia resolved, mildly distended abdomen
7	Border terrier	12	Fc	ADHAC	0.6 mg/kg q 12 h	4	1.86, 2.07	1.02	0.53	Glomerular	Glomerular	Panting resolved, mild PU/PD
8	Newfoundland	11	Me	ADHAC	1.1 mg/kg q12h	4.5	2.68 1.6	0.56	0.09	Mixed*	Physiological	Panting resolved, weight loss
9	Pinscher	11	Me	ADHAC	1.5 mg/kg q 12 h	4	9.45 9.64	4.41	6.33	Mixed	Glomerular	PU/PD resolved, improvement of polyphagia, improved alopecia, thin skin, improvement of mildly distended abdomen
17	American Staffordshire terrier	11	Fc	AIHAC	1 mg/kg q 12 h	4	5.2 5.45	0.68	0.94	Glomerular	Glomerular	PU/PD resolved, improvement of polyphagia, new symptom: circling gait

* This dog has one low molecular weight (LMW) band; therefore, the LMW band could represent prostatic protein

Mc male neutered, Me male entire, ADHAC ACTH‐dependent hyperadrenocorticism, AIHAC ACTH independent hyperadrenocorticism, PU/PD‐ polyuria and polydipsia

### Healthy dogs

All healthy dogs had a UPC < 0.2 (UPC median 0.08; min‐max, 0.06 to 0.15). Nine dogs had a single faint band at approximately 66 kDa, corresponding to albumin and one dog had no visible bands. The total urinary protein concentration of this dog was 0.04 g/L, which is below the sensitivity for the detection of protein fractions with the SDS‐AGE method used.

## DISCUSSION

This study describes the electrophoretic patterns of proteinuria in dogs with spontaneous Cushing's syndrome. Most dogs with Cushing's syndrome exhibited glomerular proteinuria and a few dogs had mixed glomerular and tubular proteinuria.

The reported incidence of pathological proteinuria in dogs with Cushing's syndrome ranges from 44 to 75% (Hurley & Vaden, 1998; Ortega et al., 1996). In our study, 15 of 19 dogs (79%) with Cushing's syndrome were proteinuric and one dog was borderline proteinuric. Although this percentage is higher than most previously reported with studies, different cut‐offs for UPC were used to define proteinuria across different studies (Caragelasco et al., 2017; Cavalcante et al., 2013; Menard et al., 2024; Smets, Lefebvre, Meij, et al., 2012). Studies employing the same cut‐offs as ours have shown a similar incidence of proteinuria (68 to 73%) (Menard et al., 2024; Smets, Lefebvre, Meij, et al., 2012). Noteworthy methods used to quantify urinary proteins and creatinine varied across studies, which could partially explain the differences. Some studies reported a higher degree of proteinuria in dogs with ADHAC. Smets, Lefebvre, Meij, et al. (2012) reported a median UPC of 1.66, with UPCs as high as 16.3. However, this study included a higher percentage of hypertensive dogs compared to our study, which could help explain the higher degree of proteinuria reported. Menard et al. (2024) did not show significant differences in blood pressure between proteinuric and non‐proteinuric dogs with ADHAC.

In our study, 12 dogs (63%) with Cushing's syndrome had glomerular proteinuria, and three dogs (16%) had mixed (glomerular and tubular) proteinuria. These findings concur with previous research on renal histopathology, showing that dogs with Cushing's syndrome predominantly have glomerular or mixed glomerular and tubular renal injury (Smets, Lefebvre, Aresu, et al., 2012; Waters et al., 1997). One study assessing the effects of exogenous glucocorticoids on renal histopathology in healthy dogs found generalised hypercellular glomerular tufts, indicative of mesangial cell proliferation, and thus glomerular changes in dogs without evidence of pre‐existing glomerular disease (Waters et al., 1997). In another study, glomerulosclerosis and tubular atrophy were identified in older dogs after administration of exogenous glucocorticoids for 16 weeks (Smets, Lefebvre, Aresu, et al., 2012). However, glomerular, tubular and interstitial changes were also present in the control group, indicating that clinically healthy, aged dogs may also have considerable renal lesions and proteinuria. Our study is also in agreement with recently published studies on SDS‐AGE urine electrophoresis (Da Riz et al., 2024; Menard et al., 2024). Menard et al. (2024) performed a study in dogs with ADHAC and proteinuria, in which a glomerular pattern was found in 10/16 (62.5%) and mixed pattern in 4/16 (25%) of dogs (Menard et al., 2024). No purely tubular pattern was identified, and in two proteinuric dogs (UPC 0.6 and 1.1), only a band corresponding to albumin was observed. In our study, one dog was proteinuric (with UPC 0.68) and exhibited a single albumin band on urine electrophoresis. This finding is most consistent with isolated albuminuria. In another study conducted by Da Riz et al. (2024), 19/26 dogs showed proteinuria on SDS‐AGE electrophoresis.

Our results are in disagreement with two previous studies on urine electrophoresis in dogs with Cushing's syndrome using SDS‐polyacrylamide gel electrophoresis (SDS‐PAGE) (Caragelasco et al., 2017; Cavalcante et al., 2013). The first study found mixed proteinuria in seven and glomerular proteinuria in only two of nine normotensive dogs with ADHAC (Caragelasco et al., 2017). In the second study, most dogs with naturally occurring Cushing's syndrome had mixed proteinuria (Cavalcante et al., 2013). The difference between these studies on one side and our findings and Menard et al. (2024) on the other side is likely because the former used SDS‐PAGE. SDS‐PAGE is more sensitive in detecting bands with very low concentrations of proteins, leading to the identification of tubular bands that are not detected using SDS‐AGE. Noteworthy, in one study using SDS‐PAGE, dogs in the control group also exhibited one to eight LMW bands (Caragelasco et al., 2017) but no tubular bands were detected in our control group using SDS‐AGE. On the other hand, the degree of renal injury in our cohort of dogs with Cushing's syndrome could be different from previous studies.

Moreover, dogs with glomerular or mixed proteinuria exhibited one to three HMW bands using SDS‐AGE, similar to findings in a previous study (Caragelasco et al., 2017) in which dogs with ADHAC had one to four HMW bands using SDS‐PAGE. However, while in our study three of 19 dogs with mixed proteinuria had one to two LMW bands, dogs with ADHAC in the previously reported study had three to eight LMW bands (Caragelasco et al., 2017).

The LMW bands detected in our study were observed between 14.3 and 26.6 kDa, while HMW bands were observed between 66 and 150 kDa. Previous studies described bands simply as LMW (<60 to 66 kDa) or HMW (>60 to 66 kDa) without specifying the approximate molecular weights. In humans, bands at 10 to 25 kDa have been associated with severe tubular damage (Bazzi et al., 2000). For example, cystatin C is a small (13 kDa) protein that can be found in the urine of dogs with renal disease. It serves as a biomarker for glomerular filtration rate (GFR) and tubular function in dogs (Ghys et al., 2014). The bands at 10 to 15 kDa may also represent β2‐microglobulin (~12 kDa) (Cobrin et al., 2013). The band at 25 to 30 kDa is a physiological finding in intact male dogs, and it is identified as a prostatic protein's arginine esterase (Schellenberg et al., 2008). The protein around the band at 26 kDa may be retinol binding protein (~22 kDa) (Loor et al., 2013) or α1‐microglobulin (Penders & Delanghe, 2004), both markers for proximal tubular dysfunction in dogs. Regarding the observed bands in the HMW range, transferrin, a protein of 76 kDa, is reported in cases of glomerular damage in dogs (Raila et al., 2007). Immunoglobulin G (150 kDa) has also been reported to be present in the urine of dogs with Cushing's syndrome (Smets, Lefebvre, Meij, et al., 2012). However, urine electrophoresis only allows identification of the size of proteins and the exact nature of the proteins cannot be inferred.

One study involving dogs with Cushing's syndrome reported a higher incidence of hypertension and albuminuria in dogs with AIHAC compared with those with ADHAC (Lien et al., 2010). In dogs with Cushing's syndrome, glomerular proteinuria may be caused by increased renal plasma flow and GFR due to haemodynamic alterations induced by glucocorticoids and hypertension (Smets et al., 2010). In our study, dogs were mostly normotensive and prehypertensive. Interestingly, UPC was slightly higher in normotensive dogs compared to prehypertensive dogs, suggesting that glucocorticoids likely influence glomerular permeability independently of blood pressure. Similar results were found in a previous study, in which no association between arterial hypertension and albuminuria or molecular weight bands was found in dogs with Cushing's syndrome (Cavalcante et al., 2013). Notably, in our study, of the three dogs with mixed proteinuria, two were prehypertensive and one was hypertensive. Although this is a small number of cases to draw any conclusion, it cannot be excluded that hypertension also affects tubular function. Another possibility is that these LMW bands were prostatic proteins (arginine esterase), since two of these three dogs were intact males. This explanation is considered unlikely because the bands were no longer detected after trilostane treatment. However, it cannot be entirely excluded, as arginine esterase is not consistently present in urine.

Some studies suggest that dogs with AIHAC may have a higher prevalence of proteinuria compared to those with ADHAC, potentially due to factors unique to adrenal tumours (e.g. tumour size, malignant behaviour or direct effects of cortisol secretion at a higher level or variability). For example, Lien et al. (2010) reported a higher incidence of hypertension and albuminuria in dogs with AIHAC compared to those with ADHAC, though the study noted no direct association between hypertension and albuminuria.

In our study, we did not observe differences in UPC or patterns of proteinuria between dogs with ADHAC and AIHAC. However, only three dogs with AIHAC were included, precluding statistical analysis.

In our study, four of seven dogs remained proteinuric after 4 to 6 months of medical treatment with trilostane and only two dogs had a decrease in UPC to <0.5. This finding is in accordance with previous studies (Caragelasco et al., 2017; Smets, Lefebvre, Meij, et al., 2012). One study reported no significant alterations in UPC during medical treatment (trilostane), with detection of HMW and LMW bands throughout the 6‐month follow‐up period (Caragelasco et al., 2017). A second study reported a decrease in UPC after treatment, but five of 13 dogs remained proteinuric even after 12 months of medical or surgical treatment (Smets, Lefebvre, Meij, et al., 2012). While our results align with existing literature, our limited sample size (only seven dogs with follow‐up examinations) and limited time of follow‐up warrants caution in generalising these findings. As dogs with worsening proteinuria exhibited elevated pre‐pill basal cortisol concentration despite adequate clinical control, it is possible that persistent proteinuria may have been due to inadequate control of their hypercortisolism. Prolonged proteinuria may also be indicative of ongoing renal dysfunction despite apparent good response to medical treatment for Cushing's syndrome. Possible mechanisms include persistent hypercortisolism, despite clinical control, inducing renal damage and/or irreversible renal lesions, such as glomerular adhesions and thickened Bowman's capsule, as described by Waters et al. (1997). It is also known that proteinuric dogs can have daily variations of UPC, especially if the UPC is above 4 (Nabity et al., 2007). In our study, dogs with Cushing syndrome and persistent proteinuria did not receive specific treatment for proteinuria during the study period (4 to 6‐months of follow‐up after diagnosis). The duration of proteinuria and long‐term effects on renal function remains unclear in these patients, as most studies evaluate outcome within a few months of initiating therapy. Further research is needed to evaluate the clinical benefit of specific treatment of proteinuria in these dogs. For now, the authors' approach is to focus on the treatment of Cushing's syndrome without specific treatment for proteinuria in most cases. If proteinuria persists despite good clinical control of Cushing syndrome, additional antiproteinuric therapy may be considered.

All dogs with pyuria (>5 WBCs/hpf) or a positive urine culture were excluded from our study. However, one dog had 4 to 8 WBCs/hpf in urine and an increase in UPC from 8.4 to 14.1 after treatment with trilostane. This dog was not excluded and, as the rest of the sediment examination was unremarkable and urine culture was negative, we assume that this was not the underlying cause for the marked increase in UPC. In addition, six of 18 dogs with Cushing's syndrome and four of 10 healthy dogs had microscopic haematuria (>5 RBCs/hpf), which can occur with cystocentesis. None of the four healthy dogs with microscopic haematuria was proteinuric and in all dogs, only a single band at 66 kDa was observed on electrophoresis. We therefore concluded that the microscopic haematuria was very unlikely to have affected the results of urine protein electrophoresis. Moreover, microscopic haematuria does not cause proteinuria in people (Tapp & Copley, 1988).

Limitations of our study include the small number of dogs with Cushing's syndrome that were available for follow‐up and the limited time of follow‐up. We acknowledge that adequate clinical control of Cushing's syndrome could have been more effectively assessed by performing a Cushing's clinical score both before and during treatment. Future studies with larger sample sizes are warranted to validate our findings. We also could not evaluate the effect of hypertension on proteinuria and electrophoretic patterns because only one dog with Cushing's syndrome was hypertensive in our study. Blood pressure measurements were performed using the oscillometric method, which may have underestimated hypertension in some dogs. We excluded prerenal and postrenal causes of proteinuria, but some renal causes cannot be completely excluded, especially in dogs with severe proteinuria, since renal biopsy was not performed. The intensity of the albumin band was not assessed, which could hinder the differentiation between pathological and low‐intensity physiological protein bands.

In conclusion, our study suggests that proteinuric dogs with Cushing's syndrome predominantly have glomerular proteinuria and occasionally mixed glomerular and tubular proteinuria. In the limited number of dogs for which follow‐up was available, proteinuria persisted in over half of the dogs 4 to 6 months after the initiation of treatment with trilostane.

### Author contributions

J. Milenkovic collected the samples, performed the study, analysed the data, interpreted the results, and wrote the first draft of the manuscript. T. Francey contributed to study design and critically reviewed the manuscript. A. Schweighauser contributed to study design and reviewed the manuscript. J. Howard contributed to study design, analysed and interpreted the data, and reviewed the manuscript. S. Kittl performed the urine bacteriological examinations. M. Campos contributed to study design, data analysis, and interpretation of the results, reviewed the manuscript, and supervised the project. All authors read and approved the final manuscript.

### Institutional Animal Care and Use Committee (IACUC) or other approval declaration

The study was approved by the Cantonal Veterinary Office of Bern, Switzerland, Nr. BE41/2021.

### Conflict of interest

The authors declare no conflicts of interest. This study was presented as an oral abstract at the 33rd ECVIM‐CA Congress.

## Funding information

The Specialisation Committee (SpezKo) of the Vetsuisse Faculty Bern.

## Data Availability

The data that support the findings of this study are available from the corresponding author upon reasonable request.

## References

[jsap70034-bib-0001] Acierno, M.J. , Brown, S. , Coleman, A.E. , Jepson, R.E. , Papich, M. , Stepien, R.L. et al. (2018) ACVIM consensus statement: guidelines for the identification, evaluation, and management of systemic hypertension in dogs and cats. Journal of Veterinary Internal Medicine, 32, 1803–1822. 30353952 10.1111/jvim.15331 PMC6271319

[jsap70034-bib-0002] Bazzi, C. , Petrini, C. , Rizza, V. , Arrigo, G. & D'Amico, G. (2000) A modern approach to selectivity of proteinura and tubulointerstitial damage in nephrotic syndrome. Kidney International, 58, 1732–1741. 11012907 10.1046/j.1523-1755.2000.00334.x

[jsap70034-bib-0003] Boretti, F. , Musella, C. , Burkhardt, W. , Kuemmerle‐Fraune, C. , Riond, B. , Reusch, C. et al. (2018) Comparison of two prepill cortisol concentration in dogs with hypercortisolism treated with trilostane. BMC Veterinary Research, 14, 417. 30591042 10.1186/s12917-018-1750-3 PMC6307252

[jsap70034-bib-0004] Caragelasco, D.S. , Kogika, M.M. , Martorelli, C.R. , Kanayama, K.K. & Simões, M.N. (2017) Urine protein electrophoresis study in dogs with pituitary‐dependent hyperadrenocorticism during therapy with trilostane. Pesquisa Veterinária Brasileira, 37, 734–740.

[jsap70034-bib-0005] Cavalcante, C.Z. , Kogika, M.M. , Bacic, A. , Santoro, M.L. , Miyashiro, S. , Saut, J.P.E. et al. (2013) Evaluation of albuminuria and electrophoresis of urinary proteins from dogs with hyperadrenocorticism and relation with systemic arterial pressure. Pesquisa Veterinária Brasileira, 33, 1357–1363.

[jsap70034-bib-0006] Cobrin, A.R. , Blois, S.L. , Kruth, S.A. , Abrams‐Ogg, A.C.G. & Dewey, C. (2013) Biomarkers in the assessment of acute and chronic kidney diseases in dog and cats. Journal of Small Animal Practice, 54, 647–655. 24152019 10.1111/jsap.12150

[jsap70034-bib-0007] Da Riz, F. , Pichard, D. , Maurey, C. , Kurtz, M. , Canonne, M. , Lavoué, R. et al. (2024) Phosphocalcic metabolism and its potential association with biomarkers of kidney disease in dogs with spontaneous hyperadrenocorticism. Veterinary Journal, 6, 106146. Available from: 10.1016/j.tvjl.2024.106146 38788995

[jsap70034-bib-0008] European Society of Veterinary Endocrinology (2021) https://www.esve.org/alive/search.aspx [accessed 13 March 2021].

[jsap70034-bib-0009] Ghys, L. , Paepe, D. , Smets, P. , Lefebvre, H. , Delanghe, J. & Daminet, S. (2014) Cystatin C: a new renal marker and its protentional use in small animal medicine. Journal of Veterinary Internal Medicine, 28, 1152–1164. 24814357 10.1111/jvim.12366 PMC4857948

[jsap70034-bib-0010] Hokamp, J.A. , Leidy, S.A. , Gaynanova, I. , Cianciolo, R.E. & Nabity, M.B. (2018) Correlation of electrophoretic urine protein banding patterns with severity of renal damage in dogs with proteinuric chronic kidney disease. Veterinary Clinical Pathology, 47, 425–434. 30125968 10.1111/vcp.12648

[jsap70034-bib-0011] Hurley, K.J. & Vaden, S.L. (1998) Evaluation of urine protein content in dogs with pituitary‐dependent hyperadrenocorticism. Journal of the American Veterinary Medical Association, 212, 369–373. 9470045

[jsap70034-bib-0012] International Renal Interest Society (IRIS) (2011) http://www.iris‐kidney.com/guidelines/staging.html [accessed 2023].

[jsap70034-bib-0013] Lees, G.E. , Brown, S.A. , Elliott, J. , Grauer, G.E. & Vaden, S.L. (2005) Assessment and management of proteinuria in dogs and cats: 2004 ACVIM forum consensus statement (small animal). Journal of Veterinary Internal Medicine, 19, 377–385. 15954557 10.1892/0891-6640(2005)19[377:aamopi]2.0.co;2

[jsap70034-bib-0014] Lien, Y.H. , Hsiang, T.Y. & Huang, H.P. (2010) Associations among systemic blood pressure, microalbuminuria and albuminuria in dogs affected with pituitary‐ and adrenal‐dependent hyperadrenocorticism. Acta Veterinaria Scandinavica, 52, 61. 21070672 10.1186/1751-0147-52-61 PMC2994942

[jsap70034-bib-0015] Loor, J.D. , Daminet, S. , Smets, P. , Maddens, B. & Meyer, E. (2013) Urinary biomarkers for acute kidney injury in dogs. Journal of Veterinary Internal Medicine, 27, 998–1010. 23952327 10.1111/jvim.12155

[jsap70034-bib-0016] Menard, M. , Kurtz, M. , Duclos, A. , Vial, J. , Maurey, C. , Cannone‐Guibert, M. et al. (2024) Description of serum symmetric dimethylarginine concentration and of urinary SDS‐AGE pattern in dogs with ACTH dependent hyperadrenocorticism. The Veterinary Journal, 305, 106108. Available from: 10.1016/j.tvjl.2024.106108 38580156

[jsap70034-bib-0017] Nabity, M.B. , Boggess, M.M. , Kashtan, C.E. & Lees, G.E. (2007) Day‐to‐day variation of the urine protein:creatinine ration in female dogs with stable glomerular proteinuria caused by X‐linked hereditary nephropathy. Journal of Veterinary Internal Medicine, 21, 425–430. 17552446 10.1892/0891-6640(2007)21[425:dvotup]2.0.co;2

[jsap70034-bib-0018] Ortega, T.M. , Feldman, E.C. , Nelson, R.W. , Willits, N. & Cowgill, L.D. (1996) Systemic arterial blood pressure and urine protein/creatinine ratio in dogs with hyperadrenocorticism. Journal of the American Veterinary Medical Association, 209, 1724–1729. 8921029

[jsap70034-bib-0019] Penders, J. & Delanghe, J.R. (2004) Alpha 1‐microglobulin: clinical laboratory aspects and applications. Clinica Chimica Acta, 356, 107–118. 10.1016/j.cccn.2004.03.037 15256311

[jsap70034-bib-0020] Raila, J. , Aupperle, H. , Raila, G. , Schoon, H.‐A. & Schweigert, F.J. (2007) Renal pathology and urinary protein excretion in a 14‐month‐old Bernese mountain dog with chronic renal failure. Journal of Veterinary Medicine. A, Physiology, Pathology, Clinical Medicine, 54, 131–135. 17381676 10.1111/j.1439-0442.2007.00919.x

[jsap70034-bib-0021] Schellenberg, S. , Mettler, M. , Gentilini, F. , Portmann, R. , Glaus, T.M. & Reusch, C.E. (2008) The effects of hydrocortisone on systemic arterial blood pressure and urinary protein excretion in dogs. Journal of Veterinary Internal Medicine, 22, 273–281. 18312279 10.1111/j.1939-1676.2007.0039.x

[jsap70034-bib-0022] Smets, P.M. , Lefebvre, H.P. , Aresu, L. , Croubels, S. , Haers, H. , Piron, K. et al. (2012) Renal function and morphology in aged beagle dogs before and after hydrocortisone administration. PLoS One, 7, e31702. 22393368 10.1371/journal.pone.0031702 PMC3290534

[jsap70034-bib-0023] Smets, P.M.Y. , Lefebvre, H.P. , Kooistra, H.S. , Meyer, E. , Croubels, S. , Maddens, B.E.J. et al. (2012) Hypercortisolism affects glomerular and tubular function in dogs. The Veterinary Journal, 192, 532–544. 21723755 10.1016/j.tvjl.2011.05.027

[jsap70034-bib-0024] Smets, P.M.Y. , Lefebvre, H.P. , Meij, B.P. , Croubels, S. , Meyer, E. , Van de Maele, I. et al. (2012) Long‐term follow‐up of renal function in dogs after treatment for ACTH‐dependent hyperadrenocorticism. Journal of Veterinary Internal Medicine, 26, 565–573. 22463105 10.1111/j.1939-1676.2012.00915.x

[jsap70034-bib-0025] Smets, P.M.Y. , Meyer, E. , Maddens, B. & Daminet, S. (2010) Cushing's syndrome, glucocorticoids and the kidney. General and Comparative Endocrinology, 169, 1–10. 20655918 10.1016/j.ygcen.2010.07.004

[jsap70034-bib-0026] Tapp, D.C. & Copley, J.B. (1988) Effect of red blood cell lysis on protein quantitation in hematuric states. American Journal of Nephrology, 8, 190–193. 3239591 10.1159/000167581

[jsap70034-bib-0027] Waters, C.B. , Adams, L.G. , Scott‐Moncrieff, J.C. , DeNicola, D.B. , Snyder, P.W. , White, M.R. et al. (1997) Effects of glucocorticoid therapy on urine protein‐to‐creatinine ratios and renal morphology in dogs. Journal of Veterinary Internal Medicine, 11, 172–177. 9183769 10.1111/j.1939-1676.1997.tb00086.x

[jsap70034-bib-0028] Zini, E. , Bonfanti, U. & Zatelli, A. (2004) Diagnostic relevance of qualitative proteinuria evaluated by use of sodium dodecyl sulfate‐agarose gel electrophoresis and comparison with renal histologic findings in dogs. American Journal of Veterinary Research, 65, 964–971. 15281656 10.2460/ajvr.2004.65.964

